# Use of the sun compass by monocularly occluded homing pigeons in a food localisation task in an outdoor arena

**DOI:** 10.1007/s10071-023-01827-5

**Published:** 2023-10-10

**Authors:** Sara Cioccarelli, Benedetta Bianchi, Dimitri Giunchi, Anna Gagliardo

**Affiliations:** Department of Biology, University of Pisa, Via Volta 6, 56126 Pisa, Italy

**Keywords:** Homing pigeons, Sun compass, Spatial learning, Visual lateralisation

## Abstract

Functional asymmetries of the avian visual system can be studied in monocularly occluded birds, as their hemispheres are largely independent. Right and left monocularly occluded homing pigeons and control birds under binocular view have been trained in a food localisation task in an octagonal outdoor arena provided with one coloured beacon on each wall. The three groups were tested after the removal of the visual beacons, so to assess their sun compass learning abilities. Pigeons using the left eye/right hemisphere system exhibited slower learning compared to the other monocular group. During the test in the arena void of visual beacons, the three groups of birds, regardless of their visual condition, were generally able to identify the training sector by exclusively relying on sun compass information. However, the directional choices of the pigeons with the left eye/right hemisphere in use were significantly affected by the removal of the beacons, while both control pigeons and birds with the right eye/left hemisphere in use displayed unaltered performances during the test. A subsample of pigeons of each group were re-trained in the octagonal arena with visual beacons present and tested after the removal of visual beacons after a 6 h fast clock-shift treatment. All birds displayed the expected deflection consistent to the sun compass use. While birds using either the left or the right visual systems were equally able to learn a sun compass-mediated spatial task, the left eye/right hemisphere visual system displayed an advantage in relying on visual beacons.

## Introduction

Functional brain lateralisation has been widely documented for several behaviours and cognitive processes in all vertebrate classes (Vallortigara and Rogers 2005; Ocklenburg and Güntürkün 2012; Güntürkün et al. 2020). Birds became important models for the study of functional lateralisation of the visual system (Daisley et al. 2009; Manns and Güntürkün 2009) because their hemispheres are largely independent. Due to the lack of the corpus callosum and the complete cross of the optic fibres, the visual inputs acquired by one eye are mainly processed by the contralateral hemisphere (Bell and Gibbs 1977; Rogers and Anson 1979; Güntürkün 1997; Güntürkün and Bugnyar 2016). For these reasons, occluding one eye and thereby excluding the contralateral brain hemisphere is a straightforward method to investigate functional asymmetry in the avian visual system (Manns and Ströckens 2014).

Among birds, pigeons display a lateralised visual system both at functional and neuroanatomical levels. The asymmetry at the neuroanatomical level occurs in the tectofugal pathway thanks to a stronger projection from the right optic tectum to the left nucleus rotundus (Güntürkün 1997). By consequence, the left entopallium is likely to better integrate input from both eyes, compared to the right entopallium. At a functional level, laboratory studies showed that the right eye/left hemisphere system has an advantage in tasks requiring categorisation and discrimination of visual stimuli (Güntürkün and Kesch 1987; Prior and Güntürkün 2001; Yamazaki et al. 2007; Rogers and Kaplan 2019).

Functional lateralisation in homing pigeons engaged in spatial tasks has been the subject of investigation in both natural and semi-natural settings (Pecchia et al. 2013). While the olfactory system is critically involved when pigeons are challenged to navigate over distant unfamiliar areas (Papi 1976; Wallraff 2005; Bonadonna and Gagliardo 2021), the visual system is importantly engaged for sun compass orientation (Schmidt-Koenig 1990) and familiar landmark-based navigation (Biro et al. 2004; Guilford and Biro 2014; Gagliardo et al. 2020).

Early displacement experiments on monocularly occluded pigeons reported either an advantage of the left hemisphere in navigation from both familiar and unfamiliar sites (Ulrich 1999; Prior et al. 2004) or a substantial lack of visual lateralisation in homing from familiar locations (Diekamp et al. 2002). More recently, a GPS tracking study on monocularly occluded pigeons showed an advantage of the right hemisphere in familiar landmark-based navigation (Pollonara et al. 2017). In fact, pigeons with the right hemisphere in use displayed a higher level of route fidelity compared to the birds with the left hemisphere in use. Interestingly, when tested in binocular conditions after clock-shift, so to set the navigational information provided by the familiar landscape in conflict with those provided by the sun compass, the birds that memorised the visual features of the overflown areas with the right hemisphere in use were more likely to re-orient on the basis of the familiar landscape. However, since the clock-shift test was conducted on pigeons in binocular condition, the question of whether one hemisphere might be specifically involved in sun compass orientation was left unanswered.

Sun compass orientation can be also studied in birds in semi-naturalistic settings, an experimental setting in which the animals could see the sky and rely on the sun compass. Pigeons are known to rely on sun compass information to localise a food reward in an outdoor arena either in the absence or presence of distinctive colour beacons placed on each sector (Bingman and Jones 1994; Gagliardo et al. 1996, 2005; Budzynski et al. 2002; Griffiths et al. 2020). The reliance on sun compass information is so strong that pigeons were shown to downgrade their reliance on visual beacons if the two kinds of information were set in conflict (Chappell and Guilford 1995). Although the relevance of sun compass mechanisms in avian spatial behaviours was widely ascertained both in arena and field (Schmidt-Koenig 1990; Sherry and Duff 1996; Guilford and Taylor 2014), brain functional asymmetries in sun compass-mediated spatial learning have been poorly investigated. Evidence of the advantage of the pigeon's left hippocampal formation in using sun compass information for localising a food reward in an octagonal outdoor arena provided with colour beacons was revealed by an experiment with birds subjected to unilateral hippocampal lesion (Gagliardo et al. 2005). Consistently, the birds with right hippocampal formation intact preferentially relied on visual beacons to localise the food reward (Gagliardo et al. 2005). It is worth noting that lesions to the left hippocampal formation affected sun compass-mediated spatial learning only when this occurred in confined conditions. An intact left hippocampal formation is a prerequisite for olfactory map learning in birds kept confined in an aviary open to winds when young pigeons need to associate wind-borne odours with the compass direction of the winds blowing at the home area (Wallraff 1966; Papi 1976; Gagliardo et al. 2001). However, hippocampal ablations, either bilateral or unilateral regardless of the side, never affected the pigeons’ ability to use the sun compass during a homing task (Gagliardo et al. 1999; Ioalè et al. 2000).

As the information provided by the sun compass mechanism are processed through the visual system, it is important to understand if there is a functional asymmetry in the pigeons’ visual pathways in sun compass-mediated spatial tasks. Recently, an experiment on monocularly occluded pigeons trained in an outdoor arena provided with visual beacons did not highlight differences in sun compass-mediated spatial learning. However, pigeons with the left eye/right hemisphere in use showed a greater reliance on visual beacons when tested after the rotation of the arena, in order to set in conflict the sun compass directional information and the position of the visual beacons (Griffiths et al. 2020). Nevertheless, this experiment leaves open the question about whether and to what extent the left or the right visual system may display an advantage in sun compass-mediated spatial tasks because the birds were not tested either in an arena void of visual beacons or in clock-shifted conditions.

Our work aimed at further investigating the occurrence of possible advantages of the left or right visual system in sun compass-based spatial learning. We trained monocularly occluded pigeons in an octagonal arena provided with distinctive visual beacons on each sector and we tested them after the removal of the beacons to assess whether the sun compass information is preferentially processed by the left or right visual system when feature cues were available. If one hemisphere is specialised in processing sun compass information during spatial learning, pigeons with the contralateral eye occluded should be unable to identify the training sector. By contrast, if both hemispheres are equally able to learn the task by relying on sun compass information, both monocular groups should be able to orient towards the training sector even in absence of visual beacons. Moreover, in order to exclude the influence of other possible uncontrolled cues and to prove their sun compass orientation abilities, a subset of pigeons was tested after a clock-shift treatment after the removal of visual beacons.

## Materials and methods

### Subjects

Thirty-four adult homing pigeons (*Columba livia*), housed in Arnino Field Station (University of Pisa, Italy) were used in the experiment. All pigeons, aged between 3 and 6 years, had previously participated in homing experiments. Throughout the duration of the experiment, the birds were housed in a mesh aviary and they had unlimited access to water. The daily ratio of food was restricted to 25 g of corn per day to maintain their motivation to perform the task. The body weight of the pigeons during the experiment varied between the 82 and 90% of their initial weight, depending on their ability to solve the task. Pigeons received their ratio of food during the experimental session. However, on days when weather conditions were unsuitable to perform the training, pigeons were fed in the aviary. Upon completion of the experiments, the subjects were moved back to their home loft with food and water ad libitum.

### Experimental groups

The three experimental groups involved in the experiment were as follows: LE/RH group (*N* = 15): pigeons trained in monocular condition with an eye cap covering the right eye, therefore with the left eye (LE) and right hemisphere (RH) in use; RE/LH group (*N* = 11): pigeons trained with an eye cap covering the left eye, therefore with the right eye (RE) and left hemisphere (LH) in use; and C group (*N* = 8): control pigeons trained in binocular condition. A conical cap with a hook Velcro® ring attached at the base was used for monocular occlusion. A complementary loop Velcro® ring was fixed through a water soluble, nontoxic glue around the selected eye after trimming out the feathers. The eye cap was applied to the pigeons before the beginning of each daily experimental session and removed when the birds were placed back in their aviary.

### Experimental apparatus

The experimental apparatus was an octagonal outdoor arena (2.6 m diameter and 0.90 m high) used in previous experiments (Chappell and Guilford 1995; Gagliardo et al. 1996, 2005; Griffiths et al. 2020). The walls of the arena were made of opaque panels, preventing the view of the surroundings, covered by a mesh net allowing the view of the sky. The arena was placed in the middle of an open field so that birds could not view external cues. Each wall of the arena had a squared hole (25 × 25 cm) giving access to a wooden box placed outside the perimeter of the structure, in which food rewards could be placed. Each of the eight boxes contained a central wooden barrier preventing the view of the food reward from inside the arena. A remotely operated release box was placed at the centre of the apparatus. At the beginning of each trial, one pigeon was placed under the release box, and after about 20 s the experimenter lifted the box by pulling a rope from outside the arena. During training, removable colour beacons 25 × 25 cm (green, black, blue, red, grey, green barred blue, yellow, orange barred white) were placed on each wall.

### Experimental procedure

The experimental protocol was divided into two subsequent experiments, each one consisting of a training phase and a test phase. The first experiment was preceded by a pretraining phase in which the birds became acquainted with the arena and the presence of food rewards inside the eight boxes.

#### Pretraining

The pretraining procedure was divided into three stages:

1. Pretraining 1: the daily food intake (25 g) was divided into eight portions that were placed inside each box ahead of the wooden barrier, so that they were visible from the centre of the arena. The session ended when the bird had eaten all the food in the eight boxes or after 30 min. This session was repeated once per day until the pigeon learn to eat the food in all the boxes.
2. Pretraining 2: the daily food intake was divided into eight portions and placed inside each box behind the wooden barrier, so that it was not visible from the centre of the arena. Therefore, the pigeon had to enter the box to see the reward. The session ended when the bird had eaten all the food available or after 30 min. This session was repeated once a day until the subject learnt to enter inside all the boxes.
3. Pretraining 3: all the food was placed only in one box, randomly assigned to each pigeon. The rewarded sector was the same for each bird throughout the whole experiment. The session consisted of 10 trials. Each trial ended when the food in the assigned box was eaten or after 30 min. During this session, the subject was allowed to enter any box until it found the rewarded box (training with correction).

If a pigeon was inactive for more than two consecutive pretraining sessions was excluded from the experiment.

#### Experiment 1

In the first experiment, the pigeons were trained to localise the food reward in the arena provided with colour beacons and tested after the removal of the visual cues. Each training session consisted of 10 trials in which the reward was placed in the sector previously assigned during pretraining 3. A trial ended when the subject entered any box or after 15 min. If the bird entered the rewarded box, it was allowed to eat all the food before being removed. If the bird entered any other box, it was removed from the arena. Training sessions were repeated until the bird reached the criterion of 24 correct choices in three consecutive sessions with at least 8 correct choices in the last session. On the day after the achievement of this learning criterion, the trained subjects were subjected to a test session after the removal of visual beacons in the arena. The test session consisted of 5 consecutive trials without food reward. Each trial ended when the bird entered a box. Pigeons inactive for more than two consecutive sessions during the training were excluded from the analysis.

#### Experiment 2

A random subsample of 6 pigeons for each group from Experiment 1 was used in Experiment 2. The three groups of pigeons were trained again to localise food in the arena provided with visual beacons, maintaining the same rewarded sector assigned in Experiment 1. The training procedures adopted in Experiment 2 were identical to those of Experiment 1, the learning criterion, however, was different because the birds had previously learnt the task. This criterion required 16 correct choices in two consecutive sessions with at least 8 correct choices in the last session. When the criterion was reached, the bird was housed for 7 days in a light-tight room for a 6 h fast clock-shift treatment (the light was switched on 6 h before sunrise and switched off 6 h before sunset). During the first 5 days of clock-shift treatment, food and water were available to the birds, while during the last two days, the birds were food-deprived. After a week inside the clock-shift room, each subject was tested in the arena without visual beacons. As in Experiment 1, the test session was composed of 5 consecutive trials without food reward and each trial ended when the bird entered a box. The pigeons were tested in the morning, between 9:00 and 13:00, which corresponds to the subjective afternoon. The expected deviation from the training direction ranged from about 90° to about 120° counter-clockwise, depending on the time of the day and on the date at which the test took place.

### Data analysis

#### Learning performances

All the statistical analyses of learning performances were performed with R 4.2.1 (R Core Team 2020).

The number of sessions needed by the three groups of pigeons to reach the criterion for Experiment 1 was compared by using the Kruskal–Wallis test, performed with the R package stats ver. 4.2.1. The Dunn Test with Bonferroni correction was used as a post hoc test to make pairwise comparisons of the three groups using the R package FSA ver. 0.9.3 (Ogle et al. 2022). An additional analysis was performed on the number of sessions needed to reach 70% of correct choices in three consecutive sessions with at least 8 correct choices in the last session in order to include also the learning performances of the pigeons that after some successful training sessions became inactive and did not make any directional choice in the arena for more than 2 consecutive sessions. Again, the Kruskal–Wallis test and Dunn’s Test with Bonferroni correction for pairwise comparisons were used to compare the learning performance of the three experimental groups.

A generalised linear mixed model (GLMM) was used to assess possible differences in task learning rates between groups. The GLMM with binomial error distribution was used to compare the number of correct choices over the total number of trials per session. The number of training sessions taken to reach the criterion, treatment and their interaction were considered in the model as fixed factors, while the subject was considered as random factor. GLMM was fitted using the R package glmmTMB ver. 1.1.4 (Brooks et al. 2017). The significance of fixed factors was tested using the Wald Chi-squared test through the R package car ver. 3.1-0 (Fox and Weisberg 2018). Model assumptions were checked with the R packages performance ver. 0.10.2 (Lüdecke et al. 2021) and DHARMa ver. 0.4.6 (Hartig and Lohse 2022). In order to assess possible differences in the slope of the learning curves post hoc pairwise comparisons were performed with the R package emmeans ver. 1.8.1–1 (Lenth et al. 2023).

##### Directional choices

The rewarded box direction was set to 0° for each bird, making the choices taken by subjects with different assigned sectors comparable. In both Experiment 1 and 2, the directional choices over the last 5 trials of the last training session and the 5 trials of the test session were used to calculate the first-order mean vector for each subject, for the training and the test, respectively. For each subject, the *V *test for circular uniformity (Batschelet 1981) was used to assess whether the directional choice distributions were different from random. In Experiment 1, the expected direction was 0°, whereas in Experiment 2, the expected direction was determined as the mean of the expected directions in the five test trials, which were computed as the difference between the sun azimuth at the real time and the sun azimuth at the subjective time.

The individual mean vectors were used to compute the second-order mean vector for each experimental group both for the training and test sessions for both Experiment 1 and 2. For each group, the One Sample Moore test (Zar 1984) was used to assess whether the individual mean vector distributions of each group were different from random. The paired Moore test (Zar 1984) was used to compare the individual mean vector distributions between training and test.

For the test session, Mardia’s two Sample test (Zar 1984) has been applied to individual mean vector distributions to make pairwise comparisons between the groups, with Bonferroni correction applied to the p values.

## Results

### Pretraining

As 6 pigeons (3 LE/RH and 3 RE/LH) remained inactive in the arena for more than 2 pretraining sessions they were excluded from the experiment. Therefore, a total of 28 pigeons (8 C, 8 RE/LH and 12 LE/RH) were involved in Experiment 1.

#### Experiment 1

**Fig. 1:**
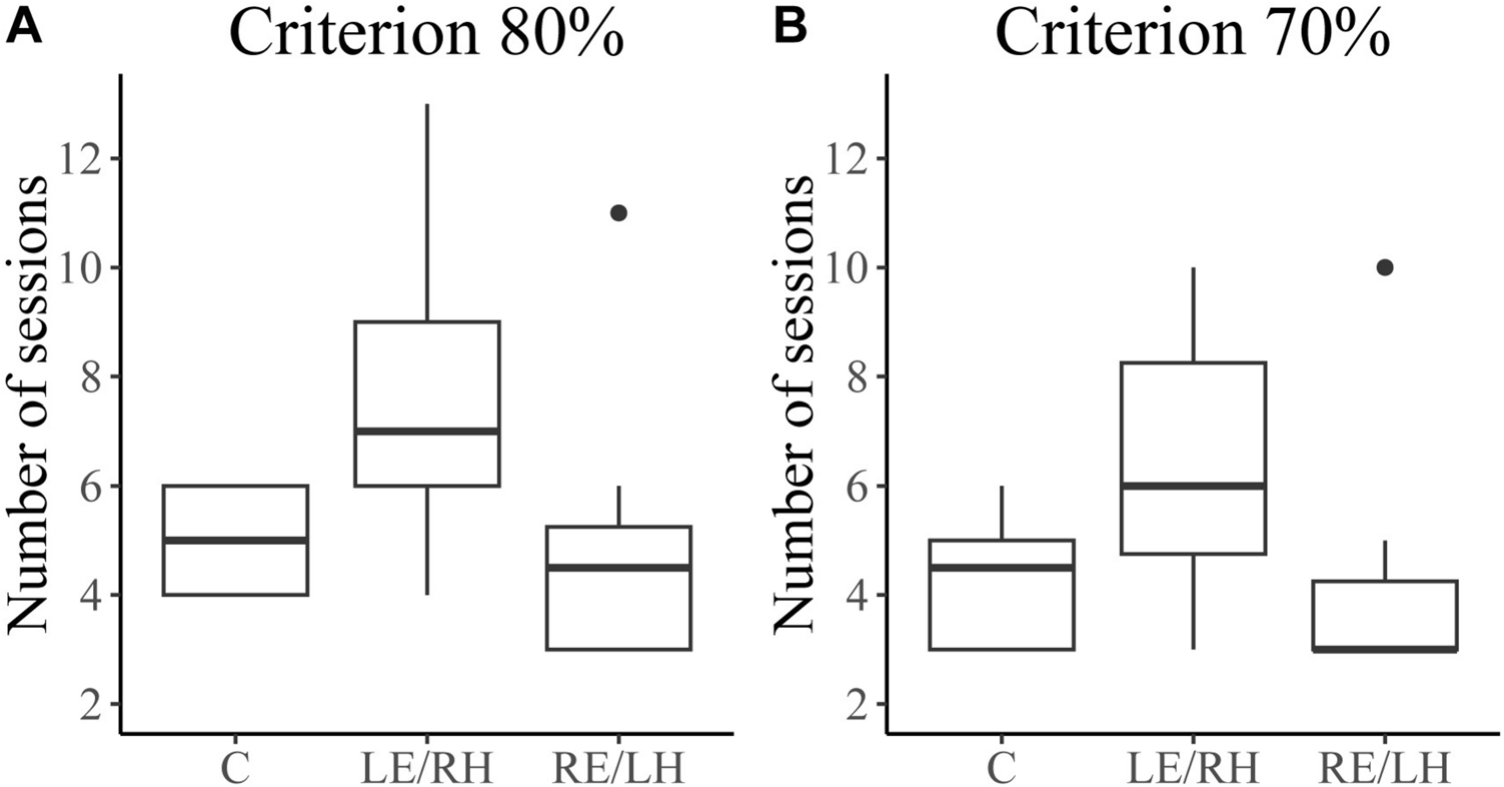
The boxplots represent the number of sessions (10 trials/session) distributions taken by each group to reach the training criterion of **a** 80% of correct choices with at least 8 correct choices in the last session (*N*_C_ = 8; *N*_LE/RH_ = 9; N_RE/LH_ = 8); **b** 70% of correct choices with at least 8 correct choices in the last session (*N*_C_ = 8; *N*_LE/RH_ = 12; *N*_RE/LH_ = 8). See Materials and method for further explanations

During learning three LE/RH pigeons became inactive in the arena after several sessions (two pigeons became inactive after 11 sessions and one animal after 9 sessions) and were excluded from the experiment. The rest of the birds (8 C, 8 RE/LH and 9 LE/RH) reached the learning criterion. However, a significant difference in the number of sessions taken by the three groups of pigeons to reach the learning criterion emerged (Kruskal–Wallis: *H*_(2)_ = 6.83, *p* = 0.03, Fig. 1a). In particular, a significant difference between the two monocular groups was found (Dunn’s Test with Bonferroni correction, LE/RH vs RE/LH: *Z* = 2.47, *p* = 0.04). By contrast, the number of sessions of control pigeons to reach the criterion is comparable to both LE/RH and RE/LH birds (C vs LE/RH: *Z* = – 1.92, *p* = 0.16; C vs RE/LH: *Z* = 0.53, *p* = 1.00).

Similar results (Kruskal–Wallis: *H*_(2)_ = 7.03, *p* = 0.03; Dunn’s Test with Bonferroni correction: LE/RH vs RE/LH: *Z* = 2.47, *p* = 0.04; C vs LE/RH: *Z* = – 1.87, *p* = 0.18; C vs RE/LH: *Z* = 0.55, *p* = 1.00) were obtained by analysing the number of sessions needed by the pigeons to reach the less restrictive criterion, 70% of correct choices in three sessions with at least 8 correct choices in the last session (Fig. 1b). Therefore, the difference between the two monocular groups persisted also by including in the analysis the 3 LE/RH birds that became inactive.

**Fig. 2:**
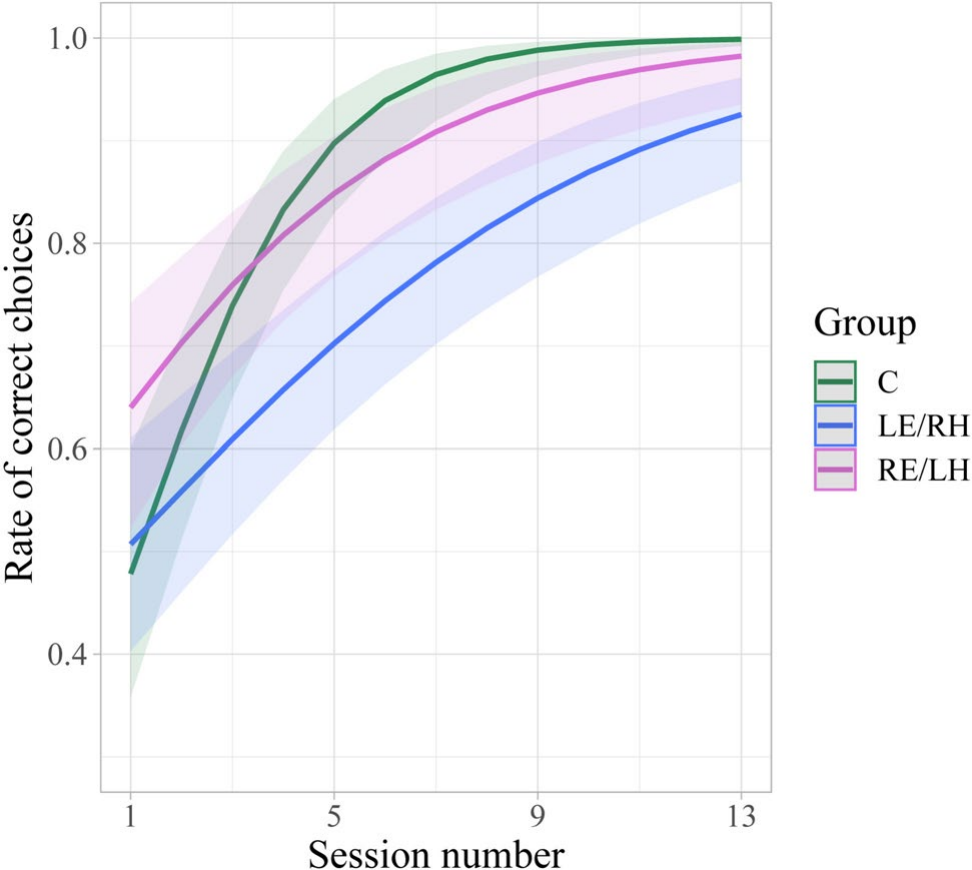
Estimated rate of correct choices (number of correct choices/session trials) of the three experimental groups against session number. The coloured lines represent the learning curve and the shaded areas represent the 95% confidence intervals from GLMM

The learning performances were also analysed by considering the percentage of correct choices per session. A significant difference between groups and the number of sessions to criterion was found (GLMM, treatment: *χ*^2^_(2)_ = 7.09, *p* = 0.03; session: *χ*^2^_(1)_ = 80.86, *p* < 0.001; interaction: *χ*^2^_(2)_ = 14.85, *p* < 0.001). The learning curve of the C group is significantly steeper than the curves of both monocular groups (emtrends, C vs LE/RH: *t*.ratio_(1,142)_ = 3.83, *p* < 0.001; C vs RE/LH: *t*.ratio_(1,142)_ = 2.62, *p* = 0.03), while the learning curves of two monocular groups showed a similar slope (LE/RH vs RE/LH: *t*.ratio_(1,142)_ = – 1.13, *p* = 0.50) (Fig. 2).

**Fig. 3:**
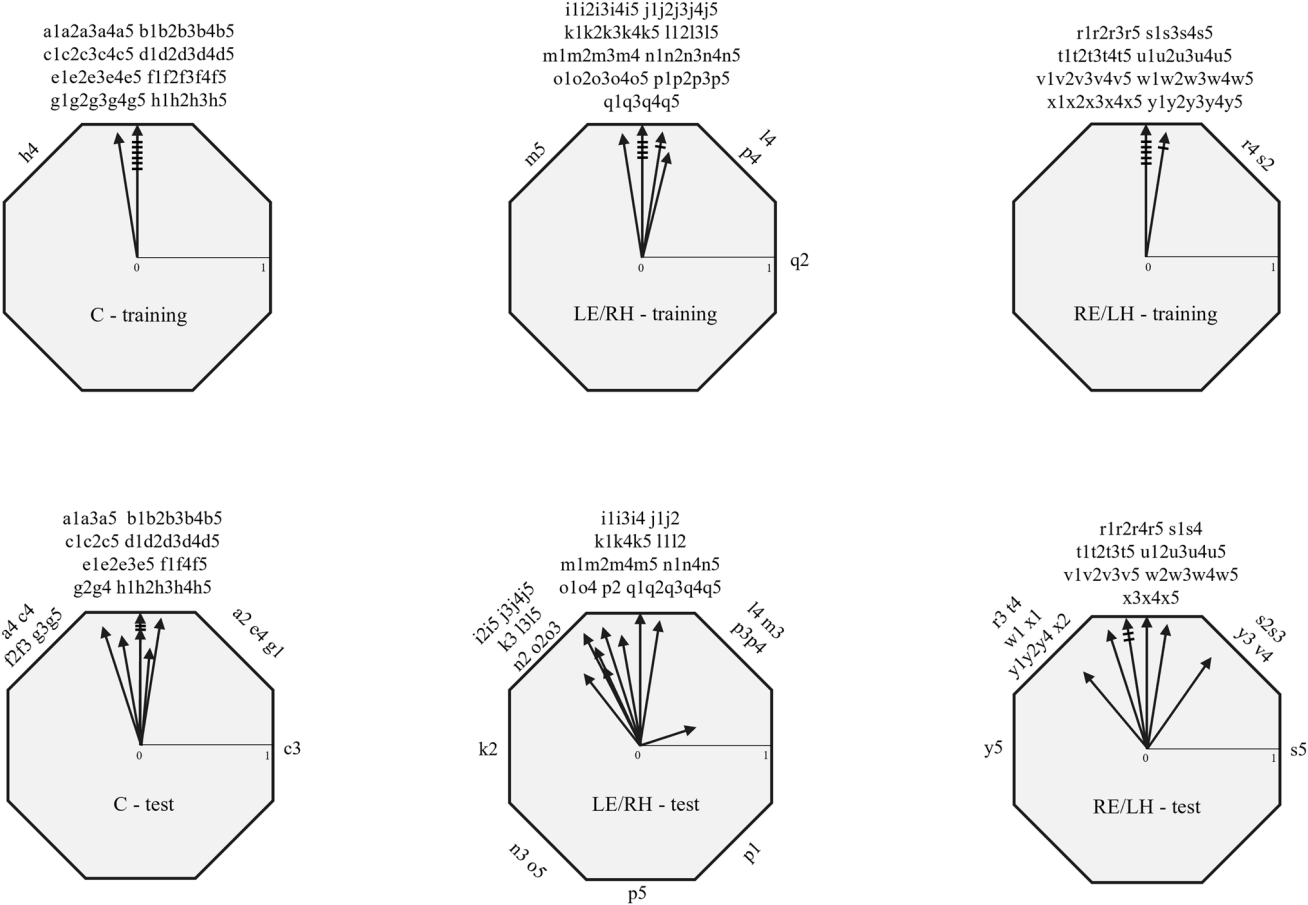
Directional choices of birds in the last 5 training trials (above) and in the 5 test trials (below) of Experiment 1. Each bird is labelled with a letter and the trial is indicated with a number (1–5). The arrows show the individual mean vectors. Lines cutting a vector represent the number of birds with the same mean vector. For each bird, the training direction is set to the top of the octagon (360°)

All the birds tested were oriented towards the training sector during both the last training and after the removal of the colour beacons in the test session, with the exception of one LE/RH pigeon (p) that, although oriented towards the rewarded sector in the training session, displayed randomly scattered choices after the removal of the colour beacons (Fig. 3, Table 1).

**Table 1:** Experiment 1

C				LE/RH				RE/LH			
id	*r*	*α*	gc	id	*r*	*α*	gc	id	*r*	*α*	gc
a	0.88	360°	+ 0.88**	i	0.93	342°	+ 0.88**	r	0.95	351°	+ 0.94***
b	1	360°	+ 1***	j	0.93	333°	+ 0.83**	s	0.84	035°	+ 0.69*
c	0.74	005°	+ 0.74**	k	0.82	335°	+ 0.74**	t	0.95	351°	+ 0.94***
d	1	360°	+ 1***	l	0.84	350°	+ 0.83**	u	1	360°	+ 1***
e	0.95	009°	+ 0.94***	m	0.95	009°	+ 0.94***	v	0.95	009°	+ 0.94***
f	0.93	342°	+ 0.88**	n	0.66	335°	+ 0.60*	w	0.95	351°	+ 0.94***
g	0.84	350°	+ 0.83**	o	0.69	322°	+ 0.54*	x	0.93	342°	+ 0.88**
h	1	360°	+ 1***	p	0.45	072°	+ 0.14	y	0.74	320°	+ 0.57*
				q	1	360°	+ 1***				

Test after removal of the visual beacons. Individual mean vectors of the birds belonging to the three experimental groups (C, LE/RH, RE/LH) computed on the basis of the five test trials are reported. The training sector was set to 360°; r, individual mean vector length; α, individual mean vector direction; gc, component towards the goal direction computed as gc = *r* (cos(*α* – *β*)) where *α* is the mean vector direction and *β* is the expected direction and corresponds to the training sector direction (360°); asterisks in the gc column represent the p values (*, **, *** corresponding to *p* < 0.05, *p* < 0.01 and *p* < 0.001, respectively) of the *V* test

The individual mean vector distributions of the three groups of pigeons were significantly different from random both in the last training and the test sessions (One sample Moore test for training phase: C, R’ = 1.59, *n* = 8, *p* < 0.001; LE/RH, *R*’ = 1.66, *n* = 9, *p* < 0.001; RE/LH, *R*’ = 1.59, *n* = 8, *p* < 0.001; One sample Moore test for test phase: C, *R*’ = 1.58, *n* = 8, *p* < 0.001; LE/RH, *R*’ = 1.58, *n* = 9, *p* < 0.001; RE/LH, *R*’ = 1.55, *n* = 8, *p* < 0.001).

Interestingly, while the control and RE/LH groups maintained the same orientation performances in the last training and the test sessions (Paired sample Moore test: C, *R*’ = 0.75, *n* = 8, *p* > 0.1; RE/LH, *R*’ = 0.94, *n* = 8, *p* > 0.1), the removal of the visual colour beacons produced a change in orientation for the pigeons with the right hemisphere in use (Paired sample Moore test: LE/RH, *R*’ = 1.28, *n* = 9, *p* < 0.01). Nevertheless, no statistical differences in orientation among the three groups after the removal of visual beacons emerged (pairwise comparisons performed with Mardia’s test with Bonferroni correction: C vs LE/RH, *U*^*2*^_8,9_ = 0.13, *p* > 0.1; C vs RE/LH, U^2^_8,8_ = 0.07, *p* > 0.1; LE/RH vs RE/LH, *U*^*2*^_9,8_ = 0.08, *p* > 0.1), confirming that all groups were able to use the sun compass information to locate the assigned sector.

#### Experiment 2

A total of 18 pigeons (6 for each group), that were previously tested in Experiment 1, participated to Experiment 2. All the pigeons used in this experiment reached the criterion, 80% of correct choices with at least 8 correct choices in the last session in the first two re-training sessions, except one RE/LH that took three sessions to reach the same level of performance. Therefore, all the pigeons showed to have a stable memory of the training sector.

**Fig. 4:**
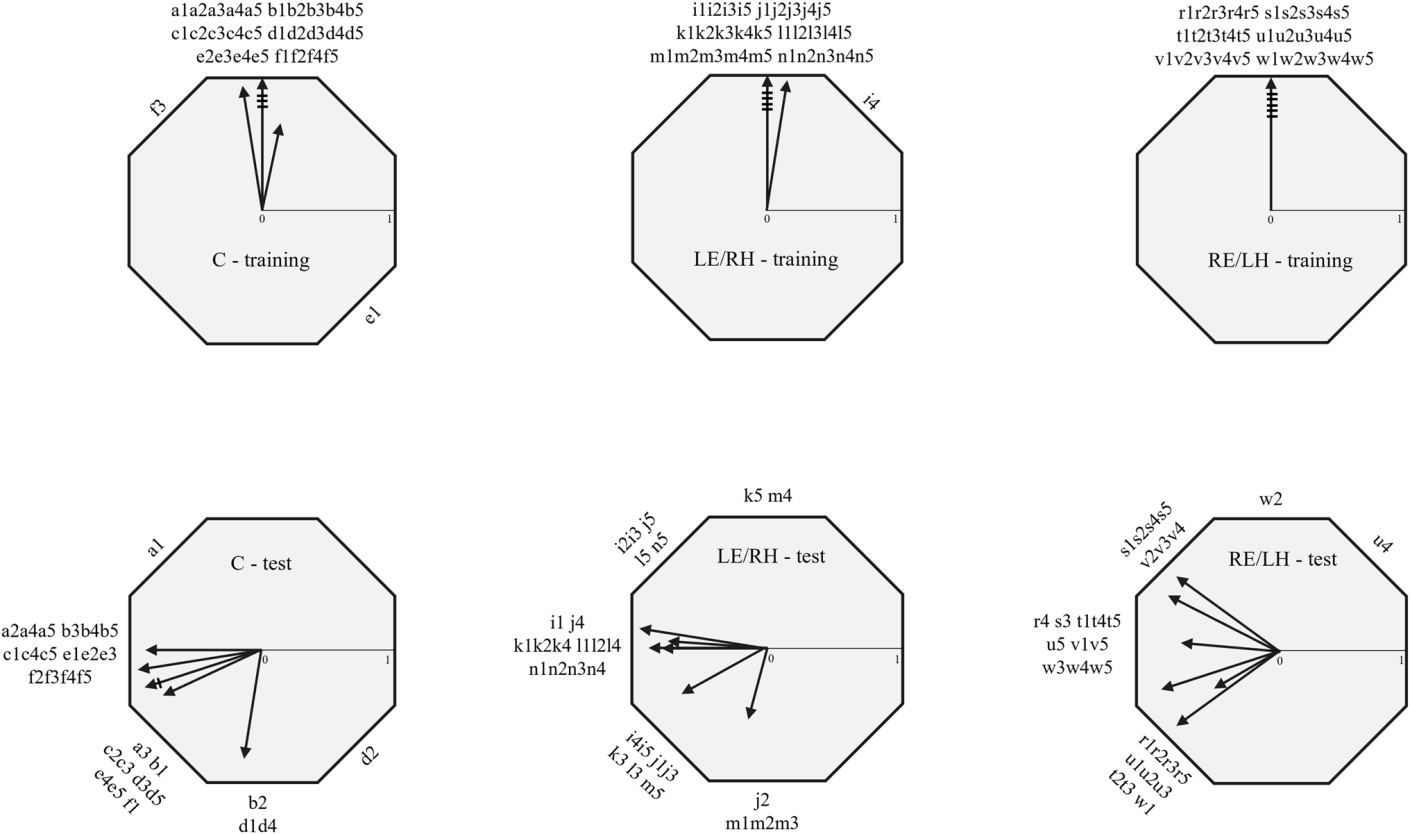
Directional choices of birds in the last 5 training trials (above) and in the 5 test trials (below) of the Experiment 2. Each bird is labelled with a letter and the trial is indicated with a number (1–5). The arrows show the individual mean vectors. Lines cutting a vector represent the number of birds with the same mean vector. For each bird, the training direction is set to the top of the octagon (360°)

All birds of the three experimental groups were oriented towards the training sector during the last training session. Five pigeons of each group were oriented towards the sector predicted by the clock-shift treatment during the test session after the removal of the colour beacons, while one bird of each group according to the V test, that keeps into account the expected direction, exhibited not oriented directional choices after the removal of beacons in the shifted condition (Fig. 4, Table 2).

**Table 2:** Experiment 2

C						LE/RH					RE/LH				
id	*r*	*α*	ed		gc	id	*r*	*α*	ed	gc	id	*r*	*α*	ed	gc
a	0.88	270°	240°		+ 0.76**	i	0.77	270°	236°	+ 0.64*	r	0.95	234°	233°	+ 0.95***
b	0.82	245°	242°		+ 0.82**	j	0.71	241°	239°	+ 0.71*	s	0.95	306°	245°	+ 0.46
c	0.93	252°	245°		+ 0.92***	k	0.74	274°	243°	+ 0.63*	t	0.93	252°	239°	+ 0.91***
d	0.84	189°	249°		+ 0.42	l	0.88	270°	248°	+ 0.82**	u	0.56	240°	255°	+ 0.54*
e	0.93	252°	247°		+ 0.93***	m	0.56	195°	249°	+ 0.33	v	0.93	297°	251°	+ 0.65*
f	0.95	261°	251°		+ 0.94***	n	0.95	279°	250°	+ 0.83**	w	0.74	275°	251°	+ 0.68*

Clock-shift test after removal of the visual beacons. Individual mean vectors of the birds belonging to the three experimental groups (C, LE/RH, RE/LH) computed on the basis of the five test trials are reported. The training sector was set to 360°; *r*, individual mean vector length; *α*, individual mean vector direction; ed, mean expected direction as predicted by the use of the sun compass; gc, component towards the goal direction computed as gc = *r* (cos(*α* – *β*)) where *α* is the mean vector direction and β is the mean expected direction as reported in ed column; asterisks in the gc column represent the *p* values (*, **, *** corresponding to *p* < 0.05, *p* < 0.01 and *p* < 0.001, respectively) of the V test. See “Materials and methods” for further explanations

**Fig. 5:**
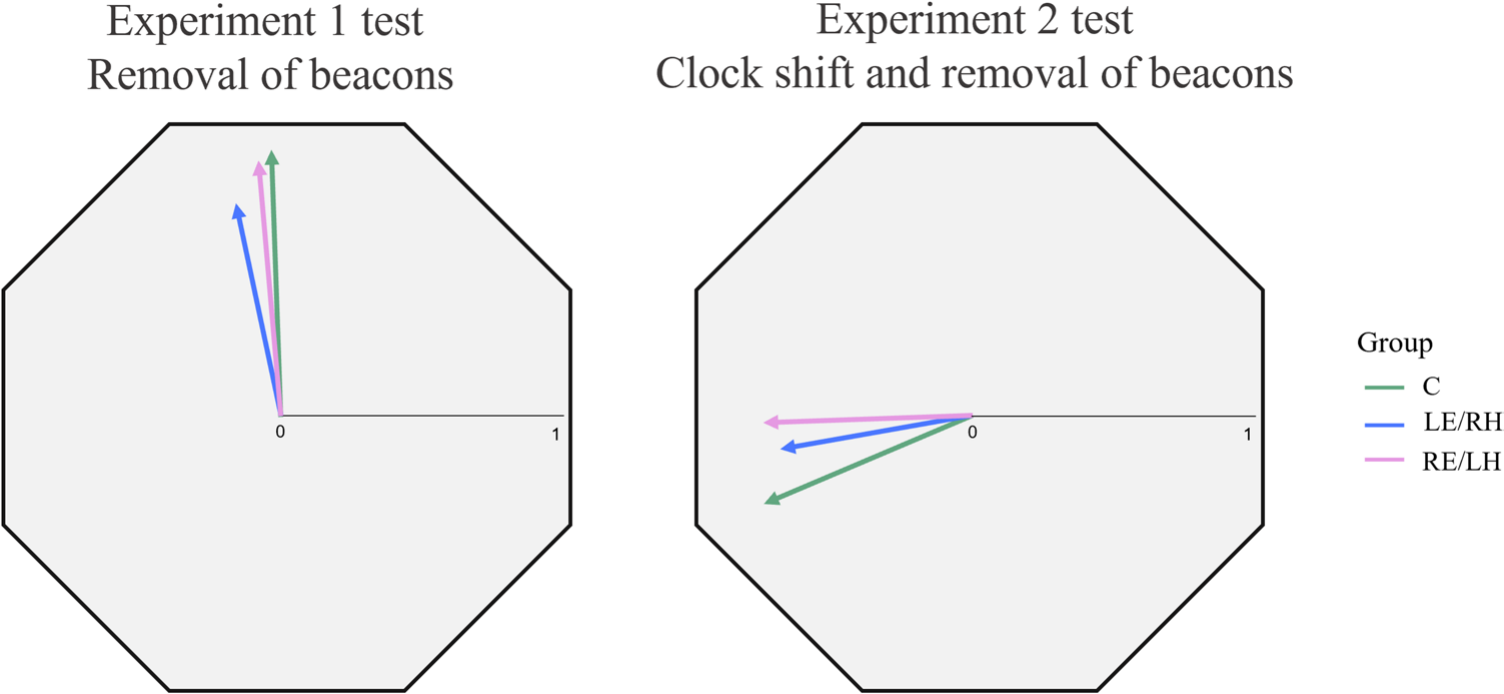
Second-order vectors of each experimental group in the first test (left) and in the second test (right). The group mean direction is indicated by the arrow and the mean vector length can be read from the scale bar. The training direction is set to the top of the octagon (360°)

The individual mean vector distributions were significantly different from random both in the last training and in the test sessions for all the experimental groups (One sample Moore test for training phase: C, *R*’ = 1.43, *n* = 6, *p* < 0.001; LE/RH, R’ = 1.43, *n* = 6, *p* < 0.001; RE/LH, *R*’ = 1.43, *n* = 6, *p* < 0.001; one sample Moore test for test phase: C, *R*’ = 1.34, *n* = 6, *p* < 0.005; LE/RH, *R*’ = 1.36, *n* = 6, *p* < 0.001; RE/LH, *R*’ = 1.24, *n* = 6, *p* < 0.01). The orientation displayed in the last training session was significantly different from the orientation displayed in the test session for all the experimental groups (Paired sample Moore test: C, *R*’ = 1.38, *n* = 6, *p* < 0.001; RE/LH, *R*’ = 1.37, *n* = 6, *p* < 0.001; LE/RH, *R*’ = 1.40, *n* = 6, *p* < 0.001). Moreover, no significant difference in the orientation of the three groups emerged (pairwise comparisons performed with Mardia’s test with Bonferroni correction: C vs LE/RH, U^2^_6,6_ = 0.18, *p* > 0.1; C vs RE/LH, U^2^_6,6_ = 0.09, *p* > 0.1; LE/RH vs RE/LH, U^2^_6,6_ = 0.06, *p* > 0.1). The impact of the clock-shift treatment on the three groups suggested that regardless of the viewing condition, the tested pigeons used the sun compass to localise the training sector after the removal of the beacons (Fig. 5).

## Discussion

In this experiment, monocularly occluded homing pigeons were trained to locate a food reward inside an octagonal outdoor arena provided with distinctive coloured beacons on each sector wall. In this condition, pigeons could rely on both sun compass information and colour beacons to localise the rewarded sector in the arena. Consistently to what was previously observed (Griffiths et al. 2020) monocularly occluded pigeons were able to learn the task as well as binocular pigeons. However, differently from what was previously reported (Griffiths et al. 2020), a significant difference between the learning performances of the two monocular groups emerged. In fact, in our experiment, the pigeons with the LE/RH in use took a significantly greater number of sessions to reach the criterion of 80% of correct choices (with at least 8 correct choices in the last session) compared to pigeons with the RE/LH in use (Figs. 1a, 2). The discrepancy between these results and what previously reported by Griffiths et al. (2020) might be due to the high number of birds excluded during the training phase (*n* = 14), whose performance has not been analysed and reported in the paper (Griffiths et al. 2020). In the present experiment, the learning performances of pigeons that were excluded by the experiment because they became inactive after several sessions (*n* = 3) were also evaluated by considering the number of sessions needed to reach a criterion of 70% of correct responses (with at least 8 correct choices in the last session). In addition, this analysis confirmed the slower learning performances of the LE/RH compared to the RE/LH birds (Fig. 1b).

During the test session, in which the coloured beacons were removed from the arena, all three experimental groups were oriented towards the training sector (Fig. 5). This suggested that both monocular groups, as well as control binocular birds, learned the position of the rewarded sector on the basis of the sun compass information. This provides clear evidence of a lack of hemispheric specialisation in the visual system in the use of the sun compass. This means that birds can learn and identify a direction regardless of the side of the visual system engaged in the task. However, the three groups of pigeons reacted differently to the removal of the coloured beacons. In fact, both the RE/LH and control pigeons displayed comparable orientation performances in the test session compared to the last training session. By contrast, the LE/RH individual orientation distribution differed between the test and the training session, due the fact that most of the pigeons in this group increased their scattering and changed their mean orientation after the removal of the beacons.

In Griffiths et al. (2020) monocularly occluded pigeons and binocular control pigeons trained to locate the food in an arena provided with visual beacons were tested in cue-shifted condition by rotating the arena 90° anti-clockwise in order to set in conflict the directional information provided by the sun compass and the positional information provided by the colour of the beacons. The LE/RH pigeons showed higher attention to featural information compared to the other monocular group (RE/LH), although the three groups oriented on the whole towards the training sector.

In our experiment, the lower learning performance during the training with coloured beacons and the lower consistency in the five test choices after the removal of the beacons displayed by the LE/RH birds might be consistent with higher attention of the right hemisphere on featural information compared to the left hemisphere. The slower learning observed in the LE/RH pigeons might reveal an engagement of the right hemisphere in processing in parallel both featural (colour beacon) and directional (sun compass information) strategies. Interestingly, control pigeons, likely to process in parallel both featural and directional cues, in the very first session of training made comparable number of errors to that of the monocularly occluded pigeons, despite that they could view with the whole visual field (Fig. 2). A similar initial impairment of learning efficiency due to parallel processing of cues has been reported in an experiment in which intact and hippocampal lesioned pigeons were trained to locate food in a rectangular arena provided with one wall of distinctive colour. The intact pigeons, able to rely on both geometrical and featural cues, were initially slower at learning compared to the hippocampal lesioned birds, unable to use geometrical information (Vargas et al. 2004).

In a recent study, homing performances of monocularly occluded pigeons were studied using GPS technology (Pollonara et al. 2017). It was observed that pigeons with the LE/RH system in use displayed an advantage in developing route fidelity compared to pigeons with the RE/LH system in use. Consistently, pigeons that had learned the route towards home with the RE/LH in use and tested in binocular conditions after a clock-shift treatment were more influenced by the phase-shift than pigeons that had learned the route with the opposite eye in use. These findings suggested a superior memory for familiar landmarks of the right hemisphere, which might be due to an advantage of the right hemisphere in processing featural information. Consistently, a lesser reliance on visual landmarks and a more consistent reliance on sun compass information could be hypothesised for the RE/LH system compared to the LE/RH system.

For the first time, the current experiment investigated the spatial behaviour of monocularly occluded pigeons after a clock-shift treatment in order to verify whether they used the sun compass to learn the training direction. Both monocular groups, as well as the control binocular group, displayed a counter-clockwise deviation as predicted by a fast clock-shift treatment (Fig. 5). This result confirmed that both monocular groups were able to learn the position of the food reward using the sun compass information.

This work pointed out the absence of hemispheric specialisation in sun compass-based spatial learning in the visual system of homing pigeons, consistent with what was reported by Griffiths et al. (2020). In summary, both left and right visual systems seem equally able to learn and use sun compass information. Nevertheless, both previous data (Pollonara et al. 2017; Griffiths et al. 2020) and these results suggest an advantage of the right hemisphere in memorising and relying on visual feature cues.

## Data Availability

The datasets generated during the current study are available from the corresponding author on reasonable request.
